# Buffalo Academy of Medicine

**Published:** 1900-02

**Authors:** Albert J. Colton


### BUFFALO ACADEMY OF MEDICINE.

REPORTED BY ALBERT J. COLTON, M. D., Secretary.

*Section on Obstetrics, Tuesday Evening, October 24, 1899.*

A REGULAR meeting of the Obstetrical Section was held October 24, 1899, at the Academy parlors.

The president, Dr. INGRAHAM, in the chair, called the meeting to order at 8.45 p. m. The minutes of the previous meeting were read and approved.

Dr. HAYD before reading his paper reported two cases with specimens of extrauterine pregnancy. Each case presenting all the symptoms usually present in a pyosalpinx. The interesting point in each case, he said was that it presented no symptoms of a ruptured tube or shock, which usually occurs in those cases. The doctor said that he had more than twenty cases and in only a very small percentage of them was he able to get history of ruptured tube; therefore it behooved one not to lay too much importance on those symptoms as indicating ectopic pregnancy. The doctor then read a very interesting and instructive paper on pus in the pelvic cavity[1].

1. See BUFFALO MEDICAL JOURNAL, December, 1899.

The paper was afterward discussed by Drs. Crockett. E. A. Smith, Lyon, Blaauw, Van Peyma, Hartwig, Breuer and Ingraham.

Dr. CROCKETT said that he had not much to add, but asserted that pus in the pelvic cavity could not be diagnosticated, but only guessed at. He said furthermore that in the majority of the cases the pus found in the pelvic cavity was not virulent. No germs of any kind were found in the largest percentage of the cases. He said it made not much difference which route was chosen, the vaginal or abdominal, if no germs were present. He said that the diagnosticating of the virulence of the pus was of great importance and recommended the withdrawing of pus with a hypodermic needle as a means of diagnosticating its virulence. Also cited a case where pus was virulent in a case operated on and patient died within twenty-four hours of septic peritonitis. The operator should select the route in which he had the greatest skill and experience.

Dr. SMITH in his discussion said that Dr. Hayd spoke with such earnestness that he must have given the subject considerable thought. The doctor said he did not see why the appendix should be omitted from the discussion of pus in the pelvic cavity from a general surgeon's point of view, although he did not care to discuss it as a gynecologist. As to the route of the operation, he would use the one that would do the least harm to his patient. He also spoke of the various means of drainage. He did not like gauze on account of its adherence to granulating surfaces and glass on account of danger of breakage, but recommended aluminum. Did not recommend breaking up adhesions unless bands interfered with the function of the bowels.

Dr. INGRAHAM asked Dr. Smith if he had any experience with hard rubber drainage tubes. The doctor had not, but intended to try them.

Dr. LYON said that marked leucocytosis indicates that inflammation exists. Also said that ascertaining the fibrin formation, which could be made at the bedside, aids in the diagnosis.

Dr. VAN PEYMA said that he had operated on a few cases where there was pus in the pelvis. He also made the statement that had before been made that Ehrlich said that leucocytosis was no indication of pus.

Dr. HARTWIG thought the future would bring about some method of suturing the tube to the vagina for drainage, so that the tubes and ovaries might be saved. As to the route of operation the cases were to be selected in accordance with its accessibility. When feasible access by the vagina should be preferred.

Dr. BREUER reported a case of multiple abscess of the pelvis where the operator was in doubt as to the route to be taken. Abscess was opened per vaginam and three days later by the abdomen. The patient died. Dr. Ingraham said in his discussion that the paper had been so exhaustive that very little was left to be said. He had operated both ways and in some cases where he had operated one way he wished that he had operated the other and vice versa. He thought that the removal of pus with hypodermic needle for diagnosis was not without danger.

In summing up Dr. Hayd thanked the members for their kind attention and the interest they had taken in the discussion. He agreed with Dr. Crockett that pus in the pelvic cavity was not ordinarily virulent. He denounced drainage. His first lessons he said were from Joseph Price, and for that reason drained his first cases and they died. With only a well-equipped laboratory, such as Kelly's, of Johns' Hopkins, can we expect to do scientific work. The doctor said that Balde in his last eighty cases had drained twice. He read from a medical journal what Morris does in appendicitis cases. Dr. Smith objected as it would require further discussion. The chair thought differently.

There were twenty-seven members present. Meeting adjourned at 10.30 p. m.

---

*Section on Surgery, Tuesday, November 7, 1899.*

REPORTED BY MARSHALL CLINTON, M. D., Secretary.

MEETING called to order by the chairman, Dr. J. HENRY DOWD. Minutes of the previous meeting were read, and, on motion, the discussion of the papers for that evening incorporated in them.

RETROPHARYNGEAL TUMOR.

Dr. MYNTER under the head of "report of cases," presented the following: "I have here the case of a large retropharyngeal tumor, pronounced by Dr. Carpenter as a spindle-celled sarcoma. The patient has had this growth in the naso-pharynx for about a year and a half and presented himself for operation about three weeks ago. As we know, these cases if allowed to progress uninterrupted by surgical procedures push their way by extension or pressure necrosis into the nose, accessory sinuses, through the cheek, or into the mouth through the palate. The methods of attacking these cases are: The intranasal route, through the cheek, after resection of the superior maxilla or as, in this case, by the way of the mouth through the soft and then hard palate. The patient being placed in the Trendelenburg position, after a preliminary tracheotomy, the uvula and soft palate were split in the median line and the soft parts reflected to either side. I think the transoral route offers the best avenue to attack anything in this region, as the hemorrhage from cases of this kind has been enormous so far as reported, and this route offers the best means of controlling the hemorrhage. By resecting the bony palate the tumor was exposed. It was found to be as large as a good sized hen's egg attached to the under side of the sphenoid bone by a narrow pedicle. The smallness of the pedicle throws doubt on the diagnosis of spindle-celled sarcoma. After removal of the tumor a staphylorrhaphy was made on the soft parts and the patient placed in bed. I show here an instrument devised some years ago by myself for the purpose of readily placing the stitches properly in this operation. The advantages in this operation are, first, absence of all external scars; second, the ease with which the hemorrhage is controlled; and, third, the field of operation being in easy sight the entire time. This man, as you can see, has made a perfect recovery.

The committee to report on the advisability of holding meetings at the different hospitals was discharged on motion, as there was no further work for them to do and no report from the committee had been received.

### ETIOLOGY AND PATHOLOGY OF APPENDICITIS.

Dr. MYNTER presented a paper on The etiology and pathology of appendicitis and their bearings on its treatment.

Before reading the paper Dr. Mynter related the conditions under which this subject was presented by him at Copenhagen. He said that today, in Denmark, the operation for appendicitis is held to be too dangerous for usual procedure, and is reserved until a hopeless case has gone too long for any benefit, and then the case is operated as a last resort. Being challenged by the medical profession to go to Denmark and defend his title paper, Dr. Mynter presented this subject on the 19th of September, 1899, before a special meeting of the Royal Society of Copenhagen. Denmark is the only country in which appendicitis is held to be a purely medical disease, and treated by drugs, viz.: opium, etc., an operation being rarely performed.

Under the head of etiology was pointed out the anatomical position, structure, and previous attacks; for all severe cases leave changes behind predisposing to fresh attacks, unless the appendix becomes destroyed by gangrene, complicated with a localised abscess, or obliterated by the destruction of the epithelium and growing together of the newly formed tissue. This later eventuality does not, however, always constitute a recovery, as it may be followed by severe gastrointestinal symptoms in the line of nervous dyspepsia, which may keep the patient in a state of chronic invalidism. Coproliths, their origin, and actions were described. The colon bacillus and the results of their varying growth and virulence in the retained secretions and congested areas was mentioned. While the author believes that at one time every case is of infectious origin, he, on the other hand, maintains that relapses may be traumatic or mechanical in their origin, and perforations and gangrene set in from pressure—necrosis from coproliths without help from the colon bacillus.

The pathology was made the basis for the classification and was given under three heads—catarrhal, infectious and ulcerative—each of which has its own peculiar symptoms, and every case may be put under one of these heads; each having its own etiology, peritoneal lesion, symptoms and prognosis. The corner stone in the pathology of appendicitis is the formation of a stricture after an appendix has been once attacked. Understanding the pathology underlying the formation of any stricture and the subsequent pathological changes which take place at the seat of the stricture, it may be very readily seen how absolutely impossible it is for any drug given by the mouth to be able to reach the sight of an inflammation in an appendix and, therefore, how impossible for medication to have an effect upon it. A drug cannot have any local action on such a condition. Therefore, the author maintained, that appendicitis is purely a surgical disease and in no respect can be considered a medical one. He compared results from numerous statistics, of the difference in treatment by medical and surgical procedures. He demonstrated that while 80 per cent. of all cases got well despite medical aid, they would have recovered simply by rest and quiet in bed. Of the remaining 20 per cent. all died under medical treatment. With their 80 per cent. the medical men delude themselves that they are able to treat this condition safely; while if the remaining 20 percentage were only handed over to the surgeons in time, the per cent of recoveries would depend absolutely on the time of the operation, the third day being the critical day in these serious cases, as 50 per cent. of these cases died if the operation was delayed beyond this time. If operated upon on or after the fourth day, almost everyone of these cases died. These cases it is understood are all ones of perforation and general septic peritonitis. If these are operated before the second day has passed from the beginning of the attack scarcely any of them die. The doctor lastly presented statistics that showed 187 cases operated by him during the last few years. Seventy-one of these had been operated since January 1, 1898, and only one had died.

DISCUSSION.

Dr. M. D. MANN said, I have studied Dr. Mynter's book on appendicitis and agree heartily with him in his remarks. The results are highly satisfactory in this disease when it is taken in time. The difficulty which presents itself is when to operate and when to wait. "When in doubt operate" is a good rule to follow. The value of one's judgment depends on the amount of work in this disease he has seen, but we can say as a general rule if any doubt lies in the mind of any one attending a case of appendicitis as to the recovery and favorable progress, cut in at once. When I say at once, I mean at once within the hour. I was called to see a woman with symptoms of a severe appendicitis and in my judgment it was necessary to operate. Within an hour from the time I saw the case I had her open and found a gangrenous appendix and a perforation in her colon. She recovered, but you can see how a few hours delay would have meant her death.

The analogy between the appendix and the Fallopian tube is very striking, when you consider their similar structure and their liability to pathological changes. There is, however, not the same tendency to gangrene and perforation or spreading inflammation in the tube that we find in the appendix. This is true of all pelvic troubles. In looking over the literature of this subject and the amount of work done on it by American surgeons, we find this country far ahead of any European country in the proper handling of this too common disease. Our statistics bear out the assertion that we are able, and do save, a much higher percentage of all cases than in any other country in the world.

Dr. BENEDICT said, I believe the surgeons are becoming more conservative in their views of the treatment of appendicitis than they were a few years ago, and that they do not, as formerly, advise immediate operation in all cases as soon as they see them. The previous rule of "operate all cases as soon as you see them" has been supplanted by a more conservative view of this disease and they are more willing to consider the views of the medical man and watch a case befoie cutting in. Cases of appendicitis, as seen clinically, are usually associated with a typhlitis or perityphlitis, as these conditions go frequently hand in hand. Recurrence is not inevitable as cases recover who never have subsequent attacks. In virulent cases associated with chronic constipation, I think the virulency is due to the action of the colon bacillus and that they are the initial cause of the lesions.

The term used this evening of nervous dyspepsia is distinctly a misnomer, as there is no such thing. A man has nervousness and dyspepsia, but not nervous dyspepsia. From the medical standpoint the question arises, Is this a septic or nonseptic case? If it be a nonseptic case or a catarrhal case do not operate, as it will recover by the aid of medication, but if on the other hand it is a septic case, it should be turned over to the surgeon for immediate operation. I consider the use of opium in these cases as both foolish and dangerous. Cathartics have a limited usefulness in some of these cases and may be used to advantage in selected cases. If we have a case of mild severity, no leucocytosis, no shock, and slight symptoms, do not operate but use an oil—a mineral one preferably and administer bismuth and salol; menthol in chronic cases may be used to advantage. The liability to typhlitis may be found in the anatomical structure of the parts, as the contents of the ileum pour into the cecum, which acts in the nature of a stand pipe. After a portion of the fluid contents are absorbed, the residue is syphoned off to the transverse colon. I ask Dr. Mynter, Is there a time not to operate?

Dr. Henderson called attention to the apparent geographical distribution of appendicitis as the number of cases seen here is greater in proportion than in New York City. The question of its relation to rheumatism might be mentioned as more cases are seen where rheumatism is common than in nonrheumatic regions. From the structure of the appendix could not it be held to be the tonsil of the abdomen? The liability of the faucil tonsils to inflammation in persons of a rheumatic diathesis suggests one possible cause of trouble in the appendix, as it may be a local manifestation of this diathesis. The concretions in the appendix and the tonsils are very similar—both are subject to catarrhal and septic inflammations.

Dr. Ingraham stated that all he knew about appendicitis he had learned by association with Dr. Mynter and in days gone by he thought that Dr. Mynter considered every one who had not had their appendix removed to be suffering from an appendicitis.

Dr. Congdon thought that the stumbling block to all was the impossibility of recognising positively between the different forms as catarrhal and septic appendicitis.

Dr. Mynter, in closing the discussion, said that may be appendicitis was rheumatic in origin or that a rheumatic diathesis had a bearing on the liability to the disease, but what difference did that make on the treatment, when the trouble had undergone the well known pathological lesions. He thinks the geographical distribution, so-called, is simply due to the fact that the cases are not recognised as appendicitis and are not treated as such. In investigating the statistics in Denmark he found the same relative proportion there that we find in this country. He does not do a laparatomy on every case of "belly-ache," as would seem from a former statement of one of the members, but his rule is, keep the patient quiet with enough opium to moderate the pain; if the patient does not begin to improve within 24 to 36 hours from the beginning of the attack, and there is still muscular retraction, pain, and the like, go in at once. All cases of every form get well if operated before 48 hours have passed since the attack began. In the worst forms all die if left until after the 4th day. The danger lies not in the operation, but in the disease. In the gangrenous forms, after the 4th or 5th day, the proper method is to make a simple incision into the abscess cavity, as the trouble will be walled off by that time, and let out the contents of the cavity. Do not in these cases go into the general peritoneal cavity. No disease in the doctor's mind is simpler to diagnosticate than an appendicitis.

In speaking of the grave lesions that may exist with slight subjective symptoms, he recalled a case that he had seen shortly at the Falls. A telephone message told him to go down there to do an appendicitis or a hysterectomy—the attending physician did not know which. He found a young woman who had been sick for a week. She was pulseless, cold, and in great shock; though mind was perfectly clear and she was not suffering from a toxemia. The belly was flat, and there was tenderness over the right ileo-cecal region. After an intravenous injection of normal saline solution the belly was opened and to his surprise he found an intussusception three feet long and totally gangrenous. A median incision was then made and the gut pulled out and the gangrenous portion resected. The mesenteric vessels was so thrombosed that when he grasped one of them he could squeeze out the clot, with which the vessel was filled, like an angle worm. The wound was left open and packed with gauze. The patient lived

36 hours and then died. This illustrates what frightful lesions may exist without the usual symptoms accompanying such a case.

Attendance, 34.

---

*Section on Pathology, Tuesday, December 19, 1899.*

Reported by FREDERICK H. MILLENER, M. D., Secretary.

THE fourth regular meeting of the Pathological Section for the season was held on Tuesday, December 19, 1899. The meeting was called to order by the secretary in the absence of the chairman, Dr. Bennett, at 8.50 p. m. On motion of Dr. Jones, Dr. Abbott was chosen as temporary chairman, and the minutes of the preceding meeting were read and corrected.

The papers of the evening were Intestinal obstruction from adherent Meckel's diverticulum, by Dr. Irving M. Snow, and The pathological condition of the middle ear, requiring radical treatment, by Dr. George F. Cott.

Dr. Irving M. Snow reported two cases and an autopsy, with specimens. The first was as follows:

Patient, a boy of three years, in good health up to the present illness. November 9th, fell a short distance from some steps, hitting himself upon abdomen, to right of umbilicus. No bad effects followed the accident. In fact there was complete relief in an hour. November 11th, the child ate quite freely of grapes and there is some reason to believe that this dietetic indiscretion was the exciting cause of the intestinal obstruction, by causing increased peristalsis and intestinal irritation. November 12th, the boy had a normal fecal passage, but commenced vomiting and vomited persistently for several days, until November 16th. During this time in spite of enemata and laxatives, the bowels remained obstinately constipated; no passage for five days. There was also present intense abdominal pain, nearly continuous, with occasional temporary relief for a few minutes. This pain was so intense as to require opiates, which was given on November 14-15-16th. When the child was asked where it hurt him, he pointed to the umbilicus. The abdomen was not tender on palpation. No tumor or induration was to be felt. The belly was slightly distended. The contents of the cecum could be plainly felt. There was gurgling and rumbling in the abdomen—only slight fever, .5 to 1.c. November 16-17th, the little patient failed visibly. Its expression was bad, face anxious and sunken; expired November 17th, 6 a. m.

Case II.—Well developed child lying in bed. Child slightly narcotised. Respiration, 12; pulse, 120; regular temperature, 99.

Slightly sighing respiration from abdominal pain; tongue slightly coated; lung, heart, skin, throat regular; rectal examination regular; no hoarseness, no abdominal tumors; liver and spleen normal; urine normal. Abdomen somewhat distended; tympanitic. Child had been nourished by enemata. Late in the afternoon the child vomited fecal matter. An injection was given; the effect of this was to cause intense abdominal pain. The child writhed with colic, vomited fecal matter and went into profound collapse. After several hours the child had small fecal stool, containing three grape seeds. The injection was followed by the passage of considerable wind and a subsidence of the abdominal swelling. Careful palpation of the relaxed abdomen revealed no tenderness nor abnormal swelling. November 18th, child apathetic, frequently sighed, occasional abdominal pain, abdomen slightly distended. Respiration, 12; temperature, 99.5°; pulse, 120. November 18-20th, same condition; after this date the pulse jerked rapidly, requiring hypodermic stimulation. There was also visible peristalsis from the 18th until death. November 22d, patient again commenced to vomit fecal matter. A quart of olive oil was given per rectum. A fecal stool, containing grape seed, followed by flatus, was the result of this procedure. No fecal matter was vomited after the enema. There was also a noticeable swelling of the abdomen. Nevertheless the patient rapidly failed, dying November 23d at 11 p. m., after an illness of twelve days. The cause of death was shock, pain and return of vomiting from intestinal obstruction. During the illness rectal alimentation was used, champagne being the only remedy retained by the stomach.

*Autopsy.*— No peritonitis; appendix healthy. The cause of the symptom was the snarling of a cord of ileum on a loop, formed by an intestinal diverticulum, attched to the ileum and connected by a small ligament to the mesentary, close to the ileo-cecal valve. More exactly stated, 12 to 14 inches from the ileo-cecal valve there projected from the ileum a glove-finger shaped process of intestine, from the free end of this diverticulum (Meckel's) proceeded a fine fibrous ligament attached to the mesentery, near the ileo-cecal valve. The diverticulum and ligament formed a loop, going one and one-half times around the ensnared intestine. In the loop Meckel's diverticulum lay uppermost, forming the upper and about one-half of the lower portion of the constricting cord. The ensnared intestine was the portion of the ileum between the insertion of the diverticulum and the ileo-cecal valve. The neck of this cord was not tightly compressed, but could be slipped to and from very easily. The lumen of the bowel was not obliterated. Above the obstruction some olive oil was floating. No fecal matter found in the intestine. Above the obstruction the intestine was slightly distended.

The case was discussed by Dr. Benedict and Dr. Williams, the latter stating that it was rare, he having only come across it once before at post mortem.

The next paper was that of Dr. Geo. F. Cott, on the

PATHOLOGICAL CONDITION OF THE MIDDLE EAR, REQUIRING RADICAL TREATMENT.

The essayist called attention to the fact that acute middle ear suppuration seldom calls for radical interference; it more often causes mastoid abscess and can readily be treated with success. The cases which are usually referred to the aurist are such as follow zymotic diseases—typhoid and scarlet fevers, diphtheria, measles, and the like. They have generally been neglected and consequently often cause caries. Some of these cases are cured by removing carious ossicles, scraping out granulation tissue, polypi, etc.

The gross anatomy was gone over and the relations to important structures, which are easily injured by operating. The different channels of infection were then mentioned. The paper closed by recommending radical treatment in all cases of caries, which cannot be relieved by all ordinary methods and in sufficient length of time.

The paper was discussed by Drs. Keiser and Howe.

Dr. Keiser stated that he concurred in the main with the view expressed by Dr. Cott, but thought that the reason the operation had not received heartier indorsement was the difficulty met with in securing the patient's consent, whose subjective symptoms were in the majority of cases comparatively nil. He thought that the indications for the operation should be summed up as follows: First, persistence of fetid discharge from the middle ear, after all other therapeutic measures have been exhausted; second, central caries or necrosis manifested by the existence of a fistulous opening over the mastoid region; third, frequently recurring mastoiditis in the course of otitis media purulenta; fourth, facial paralysis; fifth, optic neuritis, occurring during the course of middle ear disease.

Dr. Howe congratulated the writer on presenting this subject which apparently had been neglected in medical discussions, if not in practice. The radical operation, however, he considered indefinite as the simple opening of the mastoid was to a considerable extent unreliable. To be exact, the technique should be followed as first formulated by Stacke and Zaufal. This form of operation on the mastoid was probably one of the most valuable we have, not only as a cure for purulent inflammation of the middle ear, but as saving many a patient from a meningitis, with all of its dangers.

Attendance, 25.

*Section on Ophthalmology, Otology, and Laryngology, first meeting held Monday Evening, January 8, 1900.*

Reported by RICHARD H SATTERLEE, M. D., Secretary.

THE meeting was called to order at 9 p. m., Dr. W. S. RENNER in the chair.

Dr. J. C. CLEMESHA presented a case of tuberculous tumor of the nasal septum. The history was as follows:

Mary M., aged 24, unmarried, came to the Buffalo Eye and Ear Infirmary in June, 1898, for swollen eyelids and tears running over the face. The condition had then existed a year. She had treatment by an oculist without deriving any benefit. Past history negative, excepting a mild attack of typhoid fever in 1893. Her father had died some years ago from typhoid fever. Her mother in childbirth. She was the only child. Her condition at this time showed her face had broadened as compared with a photograph taken two years previously. Her eyelids somewhat thickened and show translucent folds. The alæ nasi wider and there is a slight flush over each cheek with a peculiar papular eruption. The forehead is swollen, but no pitting on pressure. Her general expression heavy and dull. The hair has become two shades darker, but is neither scanty nor dry, as is usual in myxedema. The tongue and mucous membrane of the mouth and nose show no change, though her voice has altered, having become monotonous, deliberate and slow. Her teeth have decayed rapidly during the past year. Hands always cold, the nails stunted and brittle. Temperature normal, catamenia regular; urine, specific gravity 1,023; no albumin or sugar. Examination of the neck reveals an atrophic condition of the thyroid gland, more pronounced on the right side. She was put on thyroid extract and rapidly improved, mentally and physically.

In June, 1899, she complained of inability to breathe through the right nostril, and on examination a tumor was seen growing from the cartilaginous portion of the septum. The mass was smooth, soft and grayish pink in color. It was easily removed with a snare and thought perhaps to be a myxoma. In the course of four or five months it recurred and was again removed and the tissue submitted to Dr. Gaylord of the State Pathological Laboratory for examination. The report read as follows: "The specimen removed from nasal septum proves to be a case of tuberculous infection of the membrane of the nose. There are distinct tubercles and areas of disseminated infiltration involving the connective underlying the mucous membrane proper."

Tuberculous disease of the nose is generally held to be exceedingly rare. Rice cites the fact that in 476 autopsies Willigk found one case only, and states that it may occur either as an ulceration on the nasal septum and floor and resembling tuberculous ulcerations in general, or as small papillary growths, attached to the turbinated bodies. Chiari reports six cases. He says it is a rare affection, seeing that between 1889-93 he only observed six cases out of 6,000 patients. Generally an ulcer first appears from whose edge there rapidly arise granulations that soon become confluent. The tuberculoma is red, covered with mucus or crusts with irregular bleeding surface and fairly soft. There is no pain. Later it may lead to perforation of the septum. Volkman has expressed the belief that many cases of supposed hereditary syphilis were in reality tuberculosis, while on the other hand tuberculous cases are sometimes mistaken for lupus and even malignant disease.

Tubercle of the nasal mucous membrane is generally described as occurring in the form of tumors growing from the cartilaginous septum and varying in size from a small granulation to such a bulk as would completely fill the nasal fossa. The surface is smooth or irregular, granular, covered or not with epithelium, and varying in color from a gray to a dark purple. The tumors bleed easily on examination with the probe, the inner portion being harder than the periphery. After removal they show a strong inclination to recurrence, while the site from which they have been removed indicates great reluctance to heal. Ulceration consequently results and sometimes perforation of the septum. Tuberculous ulceration of the mucous membrane sometimes appears far more intractable than the actual tumors and is generally accompanied by a purulent and sometimes fetid discharge.

There is nothing pathologically to distinguish the products of tubercular disease of the pituitary mucous membrane from the same affection elsewhere. Suffice it to say, that in the granulations and tumors, giant cells may or may not be found while the absence of bacilli is not necessarily an argument against the tuberculous nature of the growth.

The diagnosis is exceedingly difficult, seeing that the factors are so little constant. The chief diseases for which it might be mistaken are lupus and syphilis, seeing that in both of these we may have the co-existence of neoplasm and ulceration. In this connection it must not be forgotten that as lupus and syphilis may coexist so may tubercle and syphilis. The prognosis is very unsatisfactory so far as ultimate recovery is concerned. For though the tumors may be eradicated and the ulceration persuaded to heal, yet there is a strong tendency for the cicatrices to break down again. The treatment consists in the eradication of the growths and granulations, either with a sharp spoon or an electric cautery and suitable applications to the parts exposed, the treatment being conducted on rational principles, and it is highly questionable whether any of the new supposed specifics are of any service.

DISCUSSION.

Dr. E. E. Blaauw considered that a diagnosis should only be made on the clinical as well as microscopical appearances. He seemed sceptical as to the disease, being tubercular with such a family history and no other lesion.

Dr. LUCIEN HOWE inquired the differential diagnosis, and if the swelling of the lids was not due to some other condition.

Dr. F. H. MILLINER noted the perforation and inquired what other symptoms were present to determine the tubercular character of the growth, other than the findings of the microscope.

Dr. W. S. RENNER said that cases were cited by Garrish where there are localised tuberculosis with no other symptoms elsewhere. Most of these tumors undergo tubercular infiltration after operation.

Dr. CLEMESHA in summing up said that he could discover no other cause for the lids swelling. There was nothing to indicate any involvement of the optic nerve; there were no other symptoms to indicate the tubercular character of the growth; no swelling of glands elsewhere and the pharynx and lungs seemed normal.

MUSCULAR DYNAMICS.

Abstract of paper presented by Dr. Howe at the first meeting of the Ophthalmological Section was as follows:

1. Little was known of the movements of the eye previous to the middle of this century, but then a sudden impulse was given to study in that direction which within a few years gave us most of the actual facts in our possession, concerning muscular dynamics, both in a normal and in an abnormal condition.

2. These facts which were then buried, principally in Græffe's Archives of Ophthalmology, have been largely forgotten or ignored. During the last few years there has been a mass of literature relating to so-called eye strain, but few investigations based on physiological studies.

3. If we take the primary position of the two eyes as a starting point for any study we find that established fact conflicts with two respects with modern theory—namely, (*a*) That in the first position when the muscles are relaxed the visual axes are not parallel in all cases. In other words, that so-called orthophoria can only be expected in the normal eye in little over half the cases. (*b*) That the vertical meridian which theoretically should be absolutely vertical really tends to incline outward, and that this tipping outward or torsion of the axes increases with accommodation.

4. That although this principle has long been known and latterly ignored, the modern plans for its measurement are not as accurate as those established many years ago.

As an instance that many eyes are abnormal as to their muscular balance, Dr. Howe cited the test of 100 soldiers by Bannister, who concludes that the visual axes may normally tend to turn in or out, or in other words, these conditions may exist without any reflex disturbance. Bannister found a slight heterophoria in over 40 per cent. of the cases examined without any disturbance from muscular unbalance whatever. Dr. Howe wished to call attention particularly to, first, the fact that all eyes are not exactly parallel and, second, the new theories befog the real state of affairs.

Dr. BLAAUW—I think Dr. Howe is right, we must go back instead of accepting all the new theories. Most of the eye troubles in America are due to eating too rapidly. Dr. Landolt is right in saying that most eyes diverge a little bit. Some people complain when they have slight muscular defects; others do not seem to notice quite serious muscular defects.

Dr. BENNETT—I hardly think we can lay much stress on the result of Bannister's tests. Soldiers do not use their eyes for close work very much. The people who notice the muscular weakness are clerks, sewing women and people who have to use their eyes for close work. In these cases a slight defect often means considerable disturbance.

---

*Section on Medicine, Tuesday Evening, January 9, 1900.*

REPORTED BY J. C. CLEMESHA, Secretary.

THE fourth regular meeting of the Section of Medicine was held at the Academy parlors, Market Arcade, January 9, 1900. Meeting was called to order at 8.30 p. m. Dr. E. H. LONG in the chair. Minutes of the last meeting were read and approved.

ACUTE APPENDICITIS.

Dr. DELANCEY ROCHESTER read a paper on The treatment of acute appendicitis. He commenced the paper by relating the history of a case.

A girl who suffered much from chronic constipation and who was treated by him for intestinal obstrution, due to fecal impaction. During her convalescence by carelessness and disobedience to orders, she developed an acute appendicitis, which was treated by very large doses of morphia and an icebag to the abdomen. The morphia was pushed so that when sleeping her respirations were from four to six per minute. She recovered and later on during the interval her appendix was removed. The whole point in the treatment hinged on the large doses of morphia and Dr. Rochester related a case where, in a boy five years, he administered one quarter a grain of morphia every two hours for seventy-two hours.

DISCUSSION.

Dr. H. R. Hopkins said that the great difficulty was in the diagnosis of the exact condition. He had seen many die treated by the method Dr. Rochester extols, yet still believes it to be the best method in the country and at a distance from expert surgeons and well equipped hospitals. Some cases cast a heavy weight of responsibility upon the medical attendant, and while the great majority of cases do well under the Alonzo Clark method, there is a certain group of cases that inevitably die.

Dr. Eugene Smith took up the statistics of the surgical treatment. Good surgeons have a mortality of from 2 to 5 per cent., while under medical treatment 6 to 20 per cent. die. Dr. Smith considers that there are two great dangers connected with the medical treatment of acute peritonitis: (1) Gangrene and general peritonitis; (2) false hopes in regard to subsequent attacks. Dr. Smith wished to know the danger in operating in acute attacks of appendicitis.

Dr. Ingraham spoke of his early practice at a time when Dr. Alonzo Clark introduced his treatment of these conditions. He said from his own experience he did not think morphia did any good and always advised an immediate operation.

Dr. Congdon would only consider the Clark method of treatment in cases situated where it was impossible to get proper surgical aid.

Drs. Howe, Bayliss, Benedict and Thoma also discussed the paper, after which Dr. Rochester replied.

The paper Typhlitis and scolecitis, to have been read by Dr. A. L. Benedict, was put over until May 8, 1900.

Meeting adjourned 10.30 p. m. Members present, 25.

